# Tolerability and Efficacy of Topical Methotrexate in Ocular Surface Disease

**DOI:** 10.7759/cureus.80958

**Published:** 2025-03-21

**Authors:** Nathan A Seto, Calvin W Wong, Xiaowen Lu, Mitchell A Watsky, Richard W Yee

**Affiliations:** 1 Ophthalmology, MD Anderson Cancer Center, Houston, USA; 2 Ophthalmology and Visual Science, McGovern Medical School, University of Texas Health Science Center, Houston, USA; 3 Cellular Biology and Anatomy, Augusta University Medical College of Georgia, Augusta, USA; 4 Head and Neck Surgery, MD Anderson Cancer Center, Houston, USA

**Keywords:** dry eye disease (ded), inflammation of eye, ocular surface disease, prevalence of dry eye symptoms, topical methotrexate

## Abstract

Introduction: Topical immunomodulators have gained popularity in the treatment of inflammatory dry eye disease (DED). In our tertiary DED population, ocular surface inflammatory signs and symptoms refractory to conventional treatments remain a diagnostic and therapeutic challenge. This two-part in vitro toxicity and in vivo clinical study aims to determine the safety and therapeutic potential of off-label use of topical methotrexate (MTX) for the treatment of recalcitrant ocular surface disease (OSD).

Methods: Aim 1 involved an in vitro toxicity assay where primary human corneal epithelial/stromal cells (HCEs and HCSCs, respectively) were established from de-identified donor corneal rims. Epithelial cells were separated and cultured in Dulbecco’s modified Eagle’s medium (DMEM), while stromal tissue was isolated, and the cells were sub-cultured in DMEM. HCEs and HCSCs were exposed to 1 mg/mL, 2 mg/mL, or 3 mg/mL of MTX. HCEs were also treated with 10 mg/mL of MTX. Cells were photographed at 24 and 96 hours post-treatment. Aim 2 involved MTX treatment for recalcitrant surface inflammation. A retrospective chart review was conducted on patients diagnosed with recalcitrant OSD who were treated with off-label topical 1 mg/mL MTX four times a day bilaterally. The ocular surface disease index (OSDI) and symptom assessment in dry eye (SANDE) were recorded to assess symptomatic changes. Changes in objective measurements were assessed by measuring Schirmer's test, corneal fluorescein staining (CFS), and palpebral conjunctival redness (PCR). Inferential statistical analysis was performed using paired t-test and Wilcoxon signed-rank test in STATA 16.

Results: In vitro results for treated HCEs and HCSCs showed minimal changes relative to control across 1 mg/mL, 2 mg/mL, and 3 mg/mL MTX at 24 hours. Decreased cell survival of HCEs was noted after 96 hours of 1 mg/mL, 2 mg/mL, and 3 mg/mL MTX exposure. Cell survival of HCSCs at 96 hours in 1 mg/mL and 2 mg/mL MTX exposure was similar to control; however, at 3 mg/mL MTX exposure, decreased cell survival was noted. At 10 mg/mL MTX, loss of HCE cells was noted at 72 hours. Clinically, 19 patients were studied consecutively. Study times ranged from six to eight weeks, with a median follow-up time of two weeks between visits. Compared to before treatment, patients showed improvement in PCR (p=<0.01), OSDI (p=0.02), and CFS (p=0.03). SANDE and Schirmer’s 1 were also improved but not statistically significant. Visual acuity, intraocular pressure (IOP), and visual analog scale (VAS) showed no statistical change across the treatment period.

Conclusions: Topical MTX demonstrated minimal cytotoxicity in vitro and appears to be well tolerated in our patient cohort. Topical MTX may play a role in the treatment of recalcitrant DED and OSD signs and symptoms, and further study with larger sample sizes is in progress.

## Introduction

Inflammation relating to dry eye disease (DED) is understood to be both a cause and effect of DED. Inflammatory response from epithelial cells is thought to result from hyperosmolarity and instability. If the natural course of the disease is allowed to progressively worsen, inflammatory changes likewise worsen. Eventually, frank epithelial injury from the inflammatory response perpetuates the cycle of DED. When the ocular surface is inflamed, redness and swelling accompanied by complaints of pain and discomfort are common. If left untreated, ocular surface inflammation can lead to an increased risk of corneal abrasion, erosion, and ulceration. Traditional treatment of inflammatory DED has been focused on restoring a healthy tear film. More recently, targeted treatments of the associated immune response have included topical formulations of cyclosporine A, tacrolimus, and sirolimus. Biological therapies have also been developed, such as autologous serum eyedrops and interleukin-1 receptor antagonists [1,2].

Methotrexate (MTX) is a drug commonly used in rheumatology to treat rheumatoid arthritis and is also a popular chemotherapy for many types of malignancies. MTX acts as a folate analog that disrupts DNA synthesis, repair, and cellular replication. Depending on its desired use, MTX has multiple mechanisms of action. When used as a cancer treatment, MTX inhibits the reduction of folic acid into tetrahydrofolate, which inhibits DNA synthesis. When used to treat autoimmune diseases, MTX inhibits 5-aminoimidazole-4-carboxamide ribonucleotide (AICAR) transformylase, which catalyzes the final two steps in purine synthesis, leading to an increase in intracellular and extracellular adenosine [3]. Accumulation of adenosine is associated with repression of the inflammatory response [4].

In ophthalmology, MTX has already been used to treat various forms of uveitis, scleritis, ocular mucous membrane pemphigoid, and diabetic retinopathy [5]. The usual route of administration for these diseases is either oral, intravenous, or intravitreal. Intravitreal MTX has been used to treat primary intraocular lymphoma and shows potential for treating age-related macular degeneration for patients non-responsive to typical treatment [5].

Based on its applications in rheumatology and in ophthalmology as an anti-inflammatory antimetabolite, it is hypothesized that topical MTX may have therapeutic potential in patients with recalcitrant inflammatory DED based on subjective assessment, Schirmer’s 1, fluorescein staining, and palpebral redness. Thus, this work aims to first assess the safety of MTX when administered to human eye tissue (Aim 1), and secondly to evaluate the efficacy of MTX in treating inflammatory dry eye (Aim 2).

## Materials and methods

Aim 1

In this study’s in vitro toxicity assay, primary human corneal epithelial cell (HCEs) cultures were established from de-identified donor corneal rims, courtesy of Dr. Amy Estes from the Department of Ophthalmology, Medical College of Georgia, Augusta University and the Eye Guys ophthalmology practice (Augusta, GA). Methods for HCE isolation and cultivation have been previously described [6,7]. Briefly, corneal rims were washed with PBS three times, and the surrounding extra tissue and endothelium were removed under a dissecting microscope. The corneal rim tissue was incubated in Dulbecco’s modified Eagle’s medium (DMEM) (ThermoFisher Scientific, Waltham, MA) with dispase (Stemcell, Cambridge, MA) (final 1.2U/mL) at 37ºC for two hours. The epithelium was separated from the tissue and a single-cell suspension was made by gently pipetting the sheet of epithelial cells. The epithelial cell suspension was cultured in DMEM, 10% fetal bovine serum (FBS) (HyClone, Pittsburgh, PA), 1% insulin-transferrin-selenium (ITS) (ThermoFisher Scientific, Waltham, MA), 40 ug/mL gentamicin (ThermoFisher Scientific, Waltham, MA), and 5 ng/mL recombinant human epidermal growth factor (rEGF) (Gibco, #PHG0311L).

Primary human corneal stromal cell cultures (HCSC) were also established from de-identified donor corneal rims. The method for HCSC isolation and cultivation has been previously described [7,8]. Briefly, stromal tissue was cut into small pieces (approximately 0.5 mm × 0.5 mm), and after overnight digestion in DMEM containing collagenase (Sigma, St. Louis, MO) (300 μg/mL, 37 °C), the dissolved materials were collected and centrifuged at 1400 g for 2 min. The resulting pellet was re-suspended in 1.5 mL DMEM, 10% FBS, 40 ug/mL gentamicin, and 1% ITS and cultured on a 35 mm dish (Fisher Scientific, Waltham, MA) in a humidified incubator at 37 °C, 5% CO_2_. Confluent cells were passaged using 0.25% trypsin-EDTA (Sigma-Aldrich, St. Louis, MO), and sub-cultured in DMEM, 4% FBS, 1% ITS, and 40 ug/mL gentamicin. All donor-use tissue was in accordance with the Declaration of Helsinki. This study was deemed exempt by the Augusta University IRB committee.

HCEs and HCSCs were seeded onto 24-well cell culture plates (5 × 104 cells/dish), and incubated at 37 °C until cells reached 30%-40% confluence. Cells were exposed to 1 mg/mL, 2 mg/mL, or 3 mg/mL of MTX. HCEs were also treated with 10 mg/mL MTX. After treatment for 24 or 96 hours, cells were photographed using a Hoffman contrast-equipped microscope (Olympus, Tokyo, Japan) before and after rinsing off floating cells. 

Aim 2

Clinically, a retrospective chart review was performed on the off-label use of topical MTX in 19 patients with recalcitrant inflammatory DED. At the beginning of each visit, patient symptoms were assessed using the ocular surface disease index (OSDI) and symptom assessment in dry eye (SANDE). Both assessments were graded from 0-100. Objective measurements were assessed by unanesthetized Schirmer’s 1 graded 0-35 mm, corneal fluorescein staining (CFS) graded 0-3, and palpebral conjunctival redness (PCR) graded 10-100. Additionally, the following safety parameters were recorded: best corrected visual acuity (BCVA; LogMAR units), intraocular pressure (IOP; mmHg), and a visual analog scale (VAS; 0-100) that represented patient tolerability to using topical MTX. All questionnaires and clinical findings were collected by the same anterior segment fellowship-trained ophthalmologist.

Patients were instructed to use 1 mg/mL MTX in both eyes four times daily. Follow-up time ranged from six to eight weeks, with a median follow-up time of two weeks between visits. Inferential statistical analysis comparing pre- and post-treatment variables was performed in STATA 16 using paired t-test and Wilcoxon signed-rank test.

Ethics

This study followed the Declaration of Helsinki. After consultation with the Augusta University Institutional Review Board and our clinical monitor, Cherylenne Davis, it was determined that the clinical aspect of this study qualifies for an Institutional Review Board exemption under the 2018 US HHS Office of Human Research Protections (OHRP) Exemption Category 4, subpart iii.

## Results

Aim 1 involves conducting an in vitro assay

**Figure 1:**
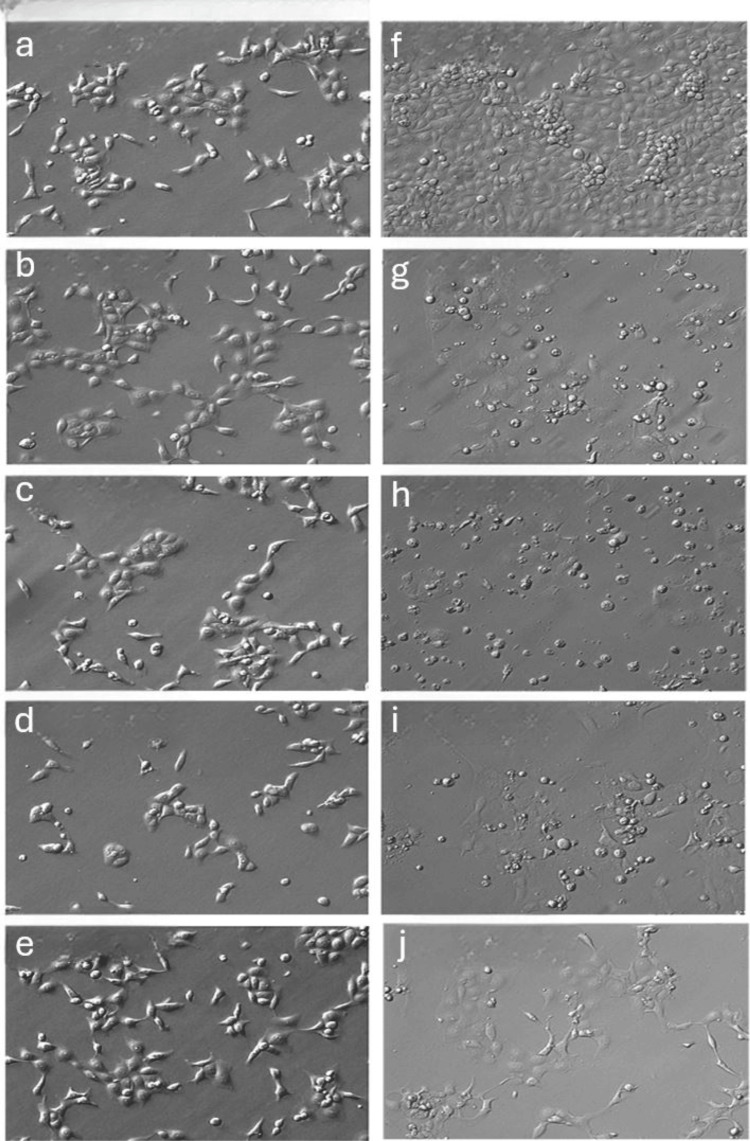
Representative in vitro assay of HCEs at varying concentrations and exposure times of MTX (a,f) Primary HCE when treated with control shows increased confluence when photographed from 24 hours to 96 hours, respectively. When photographed at 24 hours primary HCE treated with 1 mg/mL MTX (b), 2 mg/mL MTX (c), and 3 mg/mL MTX (d) show similar confluence and morphological characteristics relative to untreated control. At 96 hours of MTX exposure, primary HCE treated with 1 mg/mL MTX (g), 2 mg/mL MTX (h) and 3 mg/mL MTX (i) show apparent reduced cell density and survival. (e) When treated with 10 mg/mL MTX, primary HCE photographed at one hour remained comparable to untreated control. (j) However, at 72 hours primary HCE treated with 10 mg/mL MTX showed reduced cell density and survival. HCE: human corneal epithelium; MTX: methotrexate

**Figure 2:**
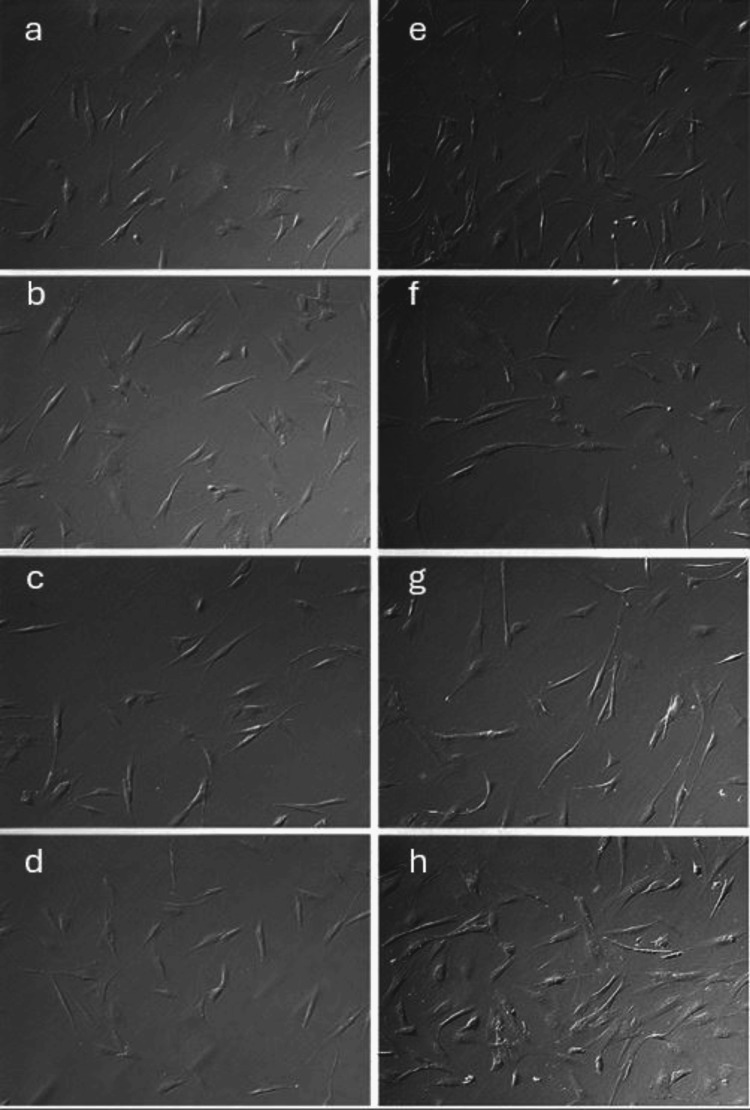
Representative in vitro assay of HCSCs at varying concentrations and exposure times of MTX (a,e) Primary HCSC treated with control photographed at 24 and 96 hours, respectively, show increased confluence as time progresses. When photographed at 24 hours, primary HCSCs treated with 1 mg/mL MTX (b), 2 mg/mL MTX (c), and 3 mg/mL MTX (d) show confluence and morphological characteristics comparable to control treatment. Similarly, at 96 hours of MTX exposure, primary HCSCs across all treatment concentrations (1 mg/mL MTX (f), 2 mg/mL MTX (g), 3 mg/mL MTX (h)) remains comparable to control treatment at 96 hours. MTX: methotrexate; HCSC: human corneal stromal cell

Treated HCE and HCSC (Figures 1, 2) showed similar morphological characteristics relative to untreated control across 1 mg/mL, 2 mg/mL, and 3 mg/mL MTX at 24 hours. Decreased cell survival of HCEs, but not HCSCs, was noted after 96 hours of 1 mg/mL, 2 mg/mL, and 3 mg/mL MTX exposure. At 10 mg/mL MTX, loss of HCE cells was noted at 72 hours.

Aim 2 involves a retrospective study of human subjects

During the course of the study, six male participants and 13 female participants were enrolled for a total of 19 participants. Age of participants ranged from 33 to 74, with a mean age of 58. Participants were studied consecutively over a period of six to eight weeks, with a median follow-up time of two weeks between visits.

For the measured safety parameters: visual acuity, IOP, and VAS of tolerability, no statistically significant change across the treatment period was observed (Table 1).

**Table 1:** Safety parameters of topical MTX (n=19) LogMAR BCVA: logarithm mean adjusted ratio score of best-corrected visual acuity. Statistical analysis was performed using a paired t-test. IOP: intraocular pressure; VAS: visual analog scale; BCVA: best corrected visual acuity, LogMAR: Logarithm of the minimum angle of resolution

	Initial mean	Final mean	Change	P-value
LogMAR BCVA	0.05 (SD=0.13)	0.0625 (SD=0.21)	0.0125	0.20
IOP (mmHg)	18.9 (SD=3.9)	16.6 (SD=2.8)	-2.25	0.27
VAS	4.47 (SD=4.7)	2.37 (SD=2.9)	-2.11	0.066

After treatment with 1 mg/mL topical MTX four times a day, statistically significant improvements in CFS (p=0.031), PCR (p=<0.01), and OSDI (p=0.02) were measured (Table 2, representing p<0.05). Statistically nonsignificant improvement was also noted for the SANDE score and Schirmer’s 1.

**Table 2:** Therapeutic parameters of topical MTX (n=19) Statistical analysis was performed using both paired t-test and Wilcoxon signed-rank test. OSDI: ocular surface disease index; SANDE: symptom assessment in dry eye; CFS: corneal fluorescein staining; PCR: palpebral conjunctival redness; MTX: methotrexate

	Initial mean	Final mean	Change	P-value
OSDI	34.4 (SD=24)	24.3 (SD=23)	-10.1	0.02
SANDE	56.9 (SD=33)	39.6 (SD=32)	-17.4	0.058
Schirmer’s 1 (mm)	10.4 (SD=7.8)	12.2 (SD=11)	1.86	0.28
CFS (5 of 19 patients that stained)	1.12 (SD=0.35)	0.375 (SD=0.52)	-0.75	0.031
PCR	59.3 (SD=14)	42.5 (SD=13)	-16.8	<0.01

## Discussion

From our in vitro results, topical MTX demonstrated an acceptable level of cytotoxicity in treated HCEs and HCSCs and thus was deemed safe enough to begin use in human eyes. Additionally, since the exposure of MTX for the in vitro results was under constant dosing when used in vivo, the low contact time of topical MTX dosing on a human eye should mitigate any potential damage to eye tissues from prolonged exposure to MTX. As mentioned above, MTX has already been administered intravitreally to treat intraocular lymphoma and other retinopathies, with studies published that outline the relative safety of intravitreal routes of MTX administration [9,10,11]. As such, when used at low concentration, (in this case 1 mg/mL), we did not anticipate or observe any major complications from the use of topical MTX, which have been discussed further below.

Prior studies in ocular surface anatomy have demonstrated that the human cornea and conjunctiva together form a united mucosal surface containing lymphoid tissues that protect the homeostasis of the ocular surface. As such, the ocular surface is considered part of the secretory immune system, containing all relevant components needed to mount a complete immune response [12,13]. Consequently, when the ocular surface is stressed from factors relating to dry eye, (e.g., increased tear film osmolarity), an inflammatory cascade response mediated by lymphoid tissue occurs [14].

Corneal epithelial cells are stimulated to activate mitogen-activated protein kinase (MAPK) pathways [15,16]. MAPKs mediate increased production and release of various pro-inflammatory cytokines, such as IL-6 and IL-8, as well as the production of matrix metalloproteinases (MMPs) [17-19]. Pathologic MMP activation may degrade corneal epithelial basal cells and proteins, leading to corneal epithelial barrier dysfunction [18]. This compromised corneal barrier leads to further increased releases of pro-inflammatory cytokines and triggers the activation of resident antigen-presenting cells (APCs). The activation of APCs leads to activation and maintenance of CD4(+) T cells [20]. Two subsets of CD4(+) T cells, Th1 and Th17, have been identified as primary drivers for desiccative stress-induced dry eye [21,22]. The accumulation of activated T cells leads to a self-perpetuating chronic immune response.

When used in a low-dose fashion, MTX inhibits AICAR transformylase, leading to the accumulation of intracellular and extracellular adenosine. Increased concentration of adenosine is associated with anti-inflammatory effects, resulting in suppression of IL-6, IL-8, and TNF-a [23]. Additionally, elevated adenosine has shown repression of T cell activation, B cell down-regulation, and increased sensitivity of activated CD95 T cells when tested in vitro with cultured human fibroblasts and endothelial cells, and in vivo with gene-knockout mice models [24-27]. This finding, coupled with the higher concentration of lymphoid tissues in the palpebral conjunctiva, may explain the improvement in PCR and in patient-reported OSDI scores [12]. The attenuation of immune response from MTX could also explain the improvement in CFS in patients who presented with punctate keratitis.

There is an active study of the relationship between PCR and ocular surface inflammation. From prior clinical observations, patients with significant inflammatory DED symptoms can present with a clear, white bulbar conjunctiva, but when their eyelids are everted, the palpebral conjunctiva can present with significant redness. The observed discordance between bulbar and PCR provides a rationale for finding treatments to directly lower the redness of the palpebral conjunctiva.

To the best of our knowledge, this study is the first to report on the use of topical MTX for inflammatory DED. As opposed to oral or intravenous administration, topical application of MTX allows a lower dose to be used to directly target the location of interest. However, topical application of MTX may still result in possible adverse effects. In our patient population, three patients reported increased redness and irritation after applying MTX. However, after stopping treatment, their complaint of redness was resolved, and redness on examination was returned to baseline within one to three days. Notably, no abnormal changes in visual acuity or IOP were measured, and based on patient responses the topical application of MTX was highly tolerable, with an initial mean VAS of 4.47 and final mean VAS of 2.37 on a scale of 0-100.

Limitations of this study include the retrospective nature of this study, the lack of a control group, and the small sample size. Larger sample size studies with a prospective design are warranted to fully elucidate the effects of topical MTX for DED. Additionally, future studies will need to explore possible overexposure effects of long-term treatment using topical MTX from our in vitro results.

## Conclusions

The findings of this two-part study suggest that topical MTX is safe to use in humans and has therapeutic potential for treating inflammatory DED. In vitro, topical MTX demonstrated an acceptable level of cytotoxicity in treated HCEs and HCSCs. In our patient cohort, topical MTX appeared to be well-tolerated. IOP, visual acuity, and VAS remained stable throughout the treatment period. Therapeutically, the retrospective chart review of patients treated with topical MTX showed statistically significant improvements in OSDI scores, PCR, and CFS. SANDE and Schirmer’s 1 scores improved but were not statistically significant at this time. Notably, the high statistical significance of PCR improvement suggests a relationship between palpebral redness and DED. Studies are currently in progress to explore the association between bulbar and palpebral hyperemia and dry eye. Overall, further studies with larger sample sizes and prospective designs are warranted to better characterize the safety and therapeutic effects of topical MTX on patient signs and symptoms for treating inflammatory DED.

## Figures and Tables

**Figure 3:**
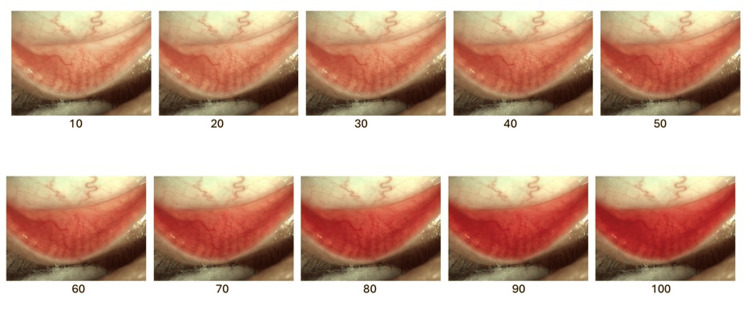
Standardized PCR scale used to assess relative injection of the patient palpebral conjunctiva The PCR scale that was developed and used here is analogous to the Validated Bulbar Redness scale developed by Schulze et. al [28]. The eyelid used in the image above was taken from a consenting individual outside of the study participants. PCR: palpebral conjunctival redness
